# Tips and Tricks in Transaortic TAVR

**DOI:** 10.21470/1678-9741-2021-0553

**Published:** 2022

**Authors:** Marco Gennari, Piero Trabattoni, Marco Agrifoglio, Gianluca Polvani, Maurizio Roberto

**Affiliations:** 1 Department of Cardiovascular Surgery, IRCCS Centro Cardiologico Monzino, Milan, Italy; 2 Department of Biomedical, Surgical, and Dental Sciences, University of Milan, Italy

**Keywords:** Transcatheter Aortic Valve Replacement, Aortic Valve Stenosis, Sternotomy, Thoracotomy, Aortic Valve Stenosis

## Abstract

Transfemoral transcatheter aortic valve replacement (TAVR) is currently the standard catheter-based treatment of severe aortic stenosis patients. Being the transfemoral route not feasible, other access sites could be chosen. Transaortic TAVR via either a J mini-sternotomy or a right anterolateral mini-thoracotomy is a good option for patients having tricky thoracoabdominal aorta. Some tips and tricks may help in getting a fast and safe transaortic procedure.

**Table:** 

Abbreviations, Acronyms & Symbols	
HOR	= Hybrid operating room
TA	= Transapical
TAo	= Transaortic
TAVR	= Transcatheter aortic valve replacement
TF	= Transfemoral

## INTRODUCTION

Transfemoral (TF) transcatheter aortic valve replacement (TAVR) is currently the most adopted technique for catheter-based aortic valve replacements. As indications for TAVR are getting broader due to current guidelines recommendations^[1]^ and positive results are being reported by randomized trials in low surgical risk patients^[2,3]^, it is crucial to offer a safe and effective alternative route when the TF route is not possible or in patients with higher risk of complications.

Transaortic (TAo) TAVR has demonstrated^[4]^ to be an efficient route when direct cannulation of the common femoral artery or navigation through the aortic arch and thoracoabdominal aorta is tricky or unsafe^[5]^, when a transapical (TA) approach is not judged the optimal strategy, and when other routes (*i.e.*, transcarotid, transaxillary, or transcaval approaches^[6]^) are not indicated.

## TECHNIQUE

**Fig. 1:**
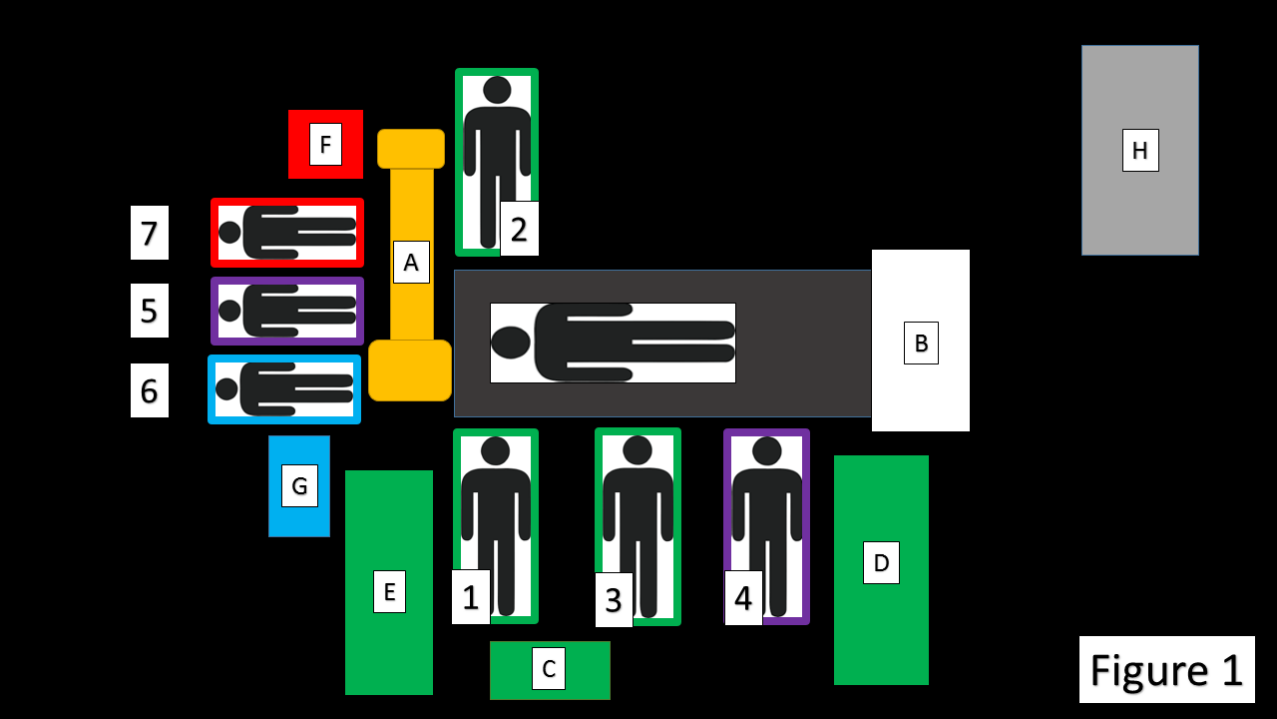
Schematic panel of the hybrid operating room setting for transaortic transcatheter aortic valve replacement (TAVR). The C-arm (A) is classically oriented as per transfemoral TAVRs, but the screen is moved to the patients’ feet (B). First operator, assistant, and eventually third operator (1, 2, and 3) are located in a standard surgical position. We routinely utilize three tables (C, D, and E) for valve preparation, surgical, and percutaneous instruments, respectively. The latter table is also useful for harboring the prepared delivery system. The nurses are at the right side of the operator (4) and at the head of the patient (5). The anesthetist (6) and, if needed, the cardiologist with an echocardiographer (7) are at the right head-side and left-head side of the patient, respectively. A running cardiopulmonary bypass (H) is present at the entrance of the operating room. F is the echo machine.

A proper hybrid operating room (HOR) setting is mandatory to perform the procedure fluently and securely. Our standard setting is depicted in Figure 1.

Briefly, the patient is lying supine, in a standard fashion. The fluoroscopic C-arm is set as per the TF route, with the machine on the patients’ left side. The screens are obliquely or transversally put at the patient’s feet. The first operator is on the right side in front of the C-arm, and the assistant is on the left side, between the C-arm and the transesophageal machine (if required). At the head of the patient, the anesthetist and the ventilator are set. We normally use three tables. The small one (Mayo) is used at the beginning and at the end and supports all the surgical instruments. The second table is used for the device preparation, and the third - put behind and to the left of the first operator - carries all the transcatheter materials and harbor the delivery catheter and valve before the TAo insertion, thus providing less distance and curve before their insertion within the delivery sheath. A preassembled and deaired cardiopulmonary bypass machine is always running just outside the HOR.

We generally get percutaneous access of the common femoral vein, with introduction of a 6-Fr short sheath and a multipolar right ventricular pacing catheter for treating potential bradyarrhythmias at incision; at this stage a half-dose of heparin is given.

A manubrotomy (J-mini-sternotomy) to the 2^nd^ or 3^rd^ right intercostal space or a right anterolateral mini-thoracotomy at the 2^nd^ intercostal space is chosen according to the length and depth of the aorta, its anatomical relationship to the sternum, and to the presence or absence of horizontal aorta (> 48°), respectively. An extension to the 4^th^ intercostal space may be needed in case of deep aorta location (> 6 cm). The pericardial sac is incised and suspended, and the remaining dose of heparin is administered.

We normally manage both the 5-F pigtail and the device delivery sheath from the ascending aorta.

Even though the non-coronary sinus is located between four and six o’clock from the operator’s view, we found easier to engage it with the pigtail catheter from the anterolateral surface of the aorta (towards the inner curvature), on which a double 4-0 proline purse-string suture is performed. A 6-Fr short sheath is transcutaneously introduced in the ascending aorta via a Seldinger technique to stabilize the catheter-sheath unit during the deployment.

**Fig. 2:**
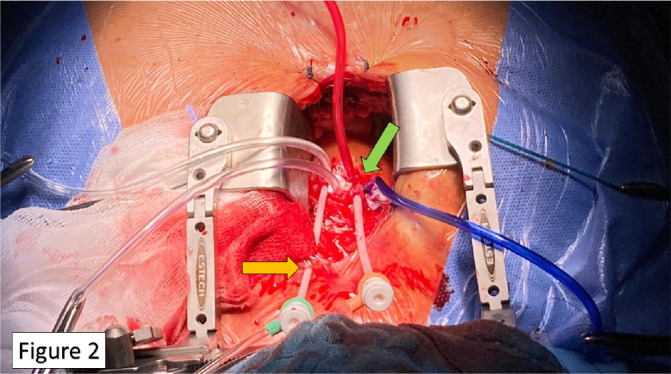
The 6-Fr sheath for the pigtail catheter is passed transcutaneously to stabilize the catheter-sheath unit (yellow arrow). Triple large purse-string sutures are performed on the ascending aorta at proper distance (green arrow).

Because of the typical tissue frailty of the elderly patients normally referred for TAVR, we generally perform three large, concentric, purse-string sutures on the outer curvature of the ascending aorta (Figure 2), ~ 2-3 cm proximal to the innominate artery, at least 1 cm distal from saphenous vein grafts or other surgical materials (*e.g.*, a Teflon felt), if present, and at least 5.5 cm away from the aortic annular plane:

- The inner purse-string is made of 3-0 braided stitch facing the assistant
- The intermediate is made of 4-0 proline, facing the operator (180° from the first suture)
- The outer is a U-shape 4-0 proline stitch facing the assistant with double Teflon felt support

**Fig. 3:**
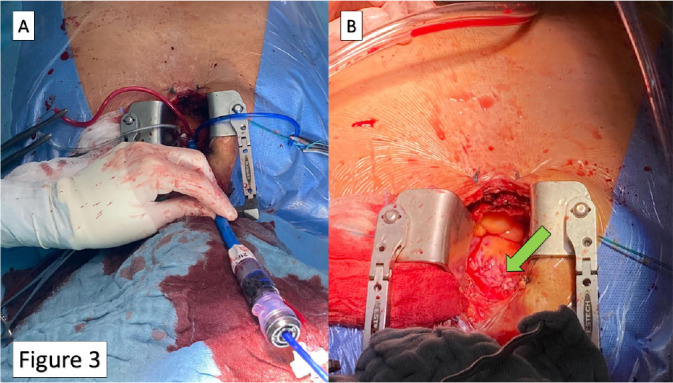
(A) Delivery sheath in place. (B) Final result after sutures tightening (green arrow).

A 7-Fr short sheath is then placed as previously described. We do not exchange it until a stiff pre-curved shape wire is within the left ventricle. Through this sheath, the valve is crossed as per operator preference; in challenging horizontal aortas, we found useful for this purpose a 3.5 to 5-Fr Judkins right catheter. We then exchange the short sheath with the delivery one (Figure 3A) to deploy the transcatheter valve in the usual manner. Finally, after sheath removal, the three sutures are tightened, and the hemostasis is evaluated (Figure 3B).

Regarding the complications of the TAo procedures, they could be addressed as general TAVR-related complications (such as cerebrovascular accidents, permanent pacemaker implantation, coronary obstruction, etc.) and TAo specific ones. The latter encompasses access site complications (aortic dissection, hematoma, laceration, bleedings) and general surgical complications (mainly infections, pericarditis, and sternal diastasis).

### The IRCCS Centro Cardiologico Monzino’s Experience

All our TAo-TAVR cases are performed in a HOR with the capability for both cardiothoracic surgical and interventional procedures, in general anesthesia and orotracheal intubation. We obtained informed consent from the patient, while our Institutional Review Board waived the need of publication consent due to the retrospective nature of this report.

In our practice, we have progressively shifted from the TA to the Tao-TAVR as an alternative access whenever the ascending aorta is suitable for harboring the introducer sheath.

Since 2016, we have adopted this route in case of unsuitable femoral access. So far, we have successfully treated 20 patients, with one operative death due to massive ischemic stroke on the 2^nd^ postoperative day.

We currently reserve the TA access mainly for cases of short or heavily atherosclerotic ascending aorta. For a proper indication of Tao-TAVR, we generally rely on high-resolution multi-detector computed tomography of the thorax. In this manner, we are able to properly estimate the ascending aorta length from the virtual basal ring and the wall’s atherosclerotic burden. Some tips and tricks on this access management could be useful for a fast procedure and favorable outcomes.

## DISCUSSION

Currently, other than TF-TAVRs account only for ≤ 15% of all the catheter-based aortic valve replacements. The main alternative routes described in the literature are: transcarotid, transaxillary, TA, and transcaval^[7]^. Also, a direct transiliac approach through a small abdominal incision has been described in highly selected patients^[8]^.

Using the ascending aorta to perform the procedure is a familiar circumstance for a cardiac surgeon, thus increasing self-confidence on the procedure. In the near future, the number of TAVR procedures will further expand; choosing the therapy with a multidisciplinary team will be more and more worthy, and different possibilities will lead to a tailored therapy.

Several observational studies have reported a comparison between TF and Tao-TAVRs results.

The first direct comparison of these two accesses made by Arai et al.^[9]^ reported similar 30-day and 1-year outcomes, but with a trend in favor of TF-TAVR. Nevertheless, the TAo approach tended to present better outcomes than the TA one.

The TAo approach have several advantages over the others. The first is no need for aortic arch crossing; in case of severe arch pathology, this may result in a reduced hazard of embolic neurological events due to debris dislodgement during wires and catheters exchanges.

Secondly, there will be only one site of operation directly controlled, with no further risk of secondary access complications. Finally, the short distance from the annulus may help in challenging situations requiring fine deployment adjustments.

Modifications of the classical TAo technique have been made in search for a less invasive direct aortic approach^[10]^, and these possibilities enrich the surgical armamentarium.

In recent years, the introduction of the so-called rapid deployment bioprosthesis has widened the possibilities to treat the aortic stenosis^[11]^. In our experience, we find them useful in the context of minimally invasive cardiac surgery and redo or multi-valvular operations. In general, once a patient is considered at moderate or higher risk for surgery and is more than 75 years old, we would treat him by TAVR.

## CONCLUSION

In conclusion, as per our experience, handling the TAo approach could be of worth for those surgeons performing TAVR, and some simple rules and tricks help in getting the procedure safe and effective.
